# Mindfulness, social evaluation anxiety, and self-regulation: exploring their association on impulsive behavior among athletes

**DOI:** 10.3389/fpsyt.2024.1404680

**Published:** 2024-05-14

**Authors:** Zhangyi Zhong, Hongyu Jiang, Huilin Wang, Yang Liu

**Affiliations:** ^1^ School of Physical Education, Hunan University of Science and Technology, Xiangtan, China; ^2^ School of Business, Hunan University of Science and Technology, Xiangtan, China; ^3^ Moray House School of Education and Sport, The University of Edinburgh, Edinburgh, United Kingdom; ^4^ School of Social and Political Science, The University of Edinburgh, Edinburgh, United Kingdom

**Keywords:** athletes, mindfulness, social evaluation anxiety, self-regulation, impulsive behavior

## Abstract

**Introduction:**

Athletes, due to frequent physical interactions in competitive sports, are prone to impulsive behavior. Impulsive behavior is a prevalent psychological factor in sports, often leading to performance-affecting errors. This cross-sectional survey investigated the relationship between mindfulness and athletes’ impulsive behavior.

**Methods:**

We sampled 403 athletes from youth training centers, universities, sports academies, and clubs in China using convenience and snowball sampling. Using AMOS v23, we analyzed the data with a structural equation model.

**Results:**

Our structural equation model confirmed that mindfulness and self-regulation inversely correlate with impulsive behavior, while social evaluation anxiety positively correlates with impulsive behavior. Furthermore, self-regulation and social evaluation anxiety serve as intermediaries in the link between mindfulness and impulsive behavior.

**Discussions:**

This research suggests introducing mindfulness meditation practices in competitive settings to improve athletes’ social evaluation anxiety and enhance their self-regulation abilities, thereby boosting their psychological health and curbing impulsive behavior.

## 1 Introduction

In recent years, impulsive behavior among athletes in the arena has become increasingly prevalent. Impulsive actions such as athletes causing harm, forfeiting matches, and displaying negative behaviors have significantly disrupted the order of sports events. These behaviors not only pose a threat to the safety of referees and spectators but also increase the likelihood of athletes sustaining injuries (1). Impulsive behavior is characterized by insufficient self-control, difficulty delaying gratification, and taking action without considering consequences (2). In the sports environment, impulsive reactions are often associated with negative emotions such as fear of failure, anger, aggressive behavior, lack of focus, anxiety, and even self-destructive tendencies (3). Impulsivity ranks among the prevalent psychological elements within the contemporary sports landscape, impacting not only athletes but also referees (4). Athletes frequently make impulsive decisions during competitions due to provocations from competitors, pressure from the audience, and referees, resulting in unconsidered actions, major technical errors, disrupted tactics, and even negative behaviors like forfeiting matches or engaging in aggressive acts in response to provocation (5).

The foremost objective for athletes in their career is to refine their abilities and achieve peak performance in their chosen sport. Achieving victory in competitive sports is the ultimate objective (6). Each athlete has their own sports career—a voluntary, multi-year commitment to sports activities aimed at reaching their peak performance in one or more sports (7). In the course of an athlete’s career, achieving outstanding results requires continuous self-improvement and resilience. However, the competitive sports environment is also filled with external stimuli that athletes must accept, process, and respond to Vaughan, Hagyard (8). These stimuli include anxiety about their own skills during regular training, reactions to evaluations and judgments from others, expectations from coaches and spectators, and pressure from competitors. These stimuli can either lead athletes to make effective decisions based on impulsive reflexes or increase their psychological stress, resulting in critical decision-making errors, a higher likelihood of technical mistakes, and ultimately, defeat, with the most apparent consequences being impulsive behaviors driven by anxiety, pressure, and anger (9).

Current studies on impulsivity within the realm of sports mainly examine the traits of impulsiveness, strategies for quantifying individual impulsiveness, and how impulsive actions influence athletes’ capabilities and performance levels. Defined as a propensity to act hastily without forethought or concern for the aftermath, impulsivity is recognized as a complex, multifaceted construct. To evaluate impulsivity, numerous assessment tools have been formulated, among which the UPPS-P Impulsive Behavior Scale is extensively applied (10). Impulsive behavior is closely linked to athletes’ skill levels and performance. High-level competitive athletes can make effective decisions based on reflexes without sacrificing accuracy (8). Conversely, competitors may engage in aggressive or negative behaviors due to factors such as fear of negative social evaluations, provocations from opponents, or pressure from referees and spectators, resulting in losses (3). Nonetheless, the exploration into the origins and consequences of impulsivity within the context of competitive athletics remains scarce. Similarly, the influence and significance of social evaluation anxiety and self-regulation in athletes have received minimal attention in scholarly research. Social evaluation anxiety, a specific type of social anxiety, tends to exacerbate impulsive actions, especially in athletes who struggle with self-regulation. Given these identified gaps in research, this investigation sets out to (1): delve deeply into the nature of impulsivity among athletes engaged in competitive sports (2); examine how mindfulness, self-regulation, and social evaluation anxiety correlate with impulsivity in athletes; and (3) offer strategies aimed at diminishing impulsivity in athletes participating in competitive arenas.

This investigation zeroes in on the impulsivity displayed by athletes in the heat of competition, probing into the connection between mindfulness and such impulsive actions. It advocates for mindfulness meditation as a strategic intervention. By reducing athletes’ social evaluation anxiety and enhancing their self-regulation, this intervention aims to reduce impulsive behavior during competitions. This research not only enriches theoretical understanding and development in related fields but also offers a fresh perspective to help improve athletes’ mindfulness levels, strengthen their self-regulation skills, and address their anxiety about social evaluation, ultimately reducing impulsive behavior and enhancing athletes’ performance.

## 2 Literature review and hypotheses

### 2.1 Concepts

#### 2.1.1 Mindfulness

Derived from Eastern Buddhist teachings, mindfulness, initially known as Sati in Sanskrit, embodies both the acute consciousness of occurrences within the experiential realm and the notion akin to memory or remembrance (11). The allure of mindfulness has surged in modern times, with scholarly pursuits progressively incorporating it into fields like alleviating anxiety, addressing mental health conditions, managing pain, supporting cancer treatments, among others (12). Characterized as an inherent aspect of personality, mindfulness involves a reasoned, impartial engagement with and acceptance of the current moment. Formal mindfulness meditation training can enhance mindfulness levels, thereby reducing anxiety, stress, impulsivity, and other negative emotions (13). It also improves emotional regulation, thereby mitigating emotional instability and enhancing self-regulation (14). For athletes, mindfulness can be understood as the capacity to observe current experiences non-judgmentally and with open-minded calmness (15). Specifically, it can be explained as “pure attention” or “present-centered awareness,” signifying a non-judgmental, non-verbal focus on the here and now (16). Mindfulness involves observing and accepting things as they are, emphasizing seeing and accepting things for what they are, rather than attempting to evaluate or change them (12). The simple phrase, “Wherever you go, there you are,” encapsulates the essence of mindfulness (15).

#### 2.1.2 Self-regulation

Self-regulation is a core component of human functioning and is a broad concept involving a dynamic process of setting an ideal end goal, taking active steps toward that goal, monitoring the process, and making adjustments as needed (17). Self-control is an integral part of self-regulation and is understood as the ability to exercise willpower or resist impulsive desires. For athletes, self-control represents the ability to resist impulsivity (18). Within self-regulation, an individual’s behavior is oriented towards an ideal end state, which includes expected behaviors, thoughts, attitudes, and emotional states. Thus, self-regulation encompasses not only behavioral but also cognitive and emotional regulation (19).

#### 2.1.3 Social evaluation anxiety

Social evaluation anxiety mainly arises from the apprehension of receiving negative judgments from others. Identified as a fundamental element of social anxiety, it results from the fear experienced in social contexts (20). Fear of negative evaluations includes fear of being judged by others, distress regarding negative evaluations by others, the desire to avoid evaluation by others, and the expectation that others will negatively evaluate oneself (21). The hallmark of social anxiety encompasses the profound dread of being judged socially, suggesting a marked positive link between social anxiety and the propensity for social evaluation (22). The anticipation of negative judgments from peers during social exchanges serves as a primary catalyst for social anxiety (23). Endler and Kocovski (24) characterized social evaluation anxiety as the distress felt in scenarios where one perceives themselves to be under scrutiny or assessment by others.

#### 2.1.4 Impulsive behavior

Impulsivity is a multidimensional concept typically characterized as a tendency to act without delaying gratification, without prior consideration, and without regard for consequences (2). Within the domain of personality traits, neuroticism stands as a crucial dimension characterized by an inclination towards negative emotional conditions, marked by elevated instances of anxiety, depression, anger, guilt, and a susceptibility to psychosomatic problems (25). In control theory terms, impulsivity can be seen as behaviors that reflect problematic tendencies not recognized when they occur, going unchecked and uncontrolled, potentially leading to severe consequences (9). For athletes, impulsivity may manifest as making instinctual and experiential breakthroughs at critical moments during a match, without excessive contemplation (26). Conversely, athletes may engage in impulsive behaviors such as violence, negative play, forfeiting matches, or other impulsive actions in response to pressure or provocations from opponents, even influencing referees during matches (27).

### 2.2 Hypotheses

#### 2.2.1 Mindfulness, social evaluation anxiety, and self-regulation

Current studies have shown that mindfulness exerts a considerable influence on social anxiety (28, 29), subjective well-being (30), the management of emotions (14), and clinical intervention (31). The influence of mindfulness on social evaluation anxiety primarily revolves around changing emotional responses through the alteration of cognitive-emotional processes, thus reducing individual stress (32). Additionally, it improves psychological health by increasing subjective well-being, reducing psychological symptoms, and enhancing emotional responses (33).

Furthermore, the level of mindfulness has a significant impact on self-regulation, mainly by enhancing emotional regulation and delaying gratification abilities (34). Studies suggest that mindfulness can reduce factors interfering with self-regulation, promote compliance with medical advice, and provide individuals with higher mindfulness levels with more self-regulation resources for self-reflection and repair, thereby alleviating stress and reducing impulsive behavior (35). People with higher mindfulness levels can more readily accept themselves, which is particularly beneficial for athletes in acknowledging their strengths and weaknesses in terms of physical appearance and technical skills.

Negative assessments are closely linked to depression, anxiety, and stress, and their intensity often grows with aging and the accumulation of social experiences (36). Although a gradual escalation in the apprehension of negative evaluations is a typical aspect of an athlete’s career progression, these fears may intensify and persist over time instead of diminishing (37). After a period of accumulating fears of negative evaluation, individuals may exhibit more externalized issues (38). Individuals with social anxiety can lower their anxiety levels and improve their mental health through self-affirming self-regulation skills (39). Therefore, this study proposes the following hypotheses:

Hypothesis 1 (H1): Mindfulness is negatively correlated with social evaluation anxiety.
Hypothesis 2 (H2): Mindfulness is positively correlated with self-regulation.
Hypothesis 3 (H3): Self-regulation is negatively correlated with social evaluation anxiety.

#### 2.2.2 Social evaluation anxiety, self-regulation, and impulsive behavior

Existing studies indicate that the experience of social evaluation anxiety in individuals can result in feelings such as anger, self-doubt, and anxiety due to negative societal judgments (40). After accumulating over time, this anxiety can lead to a series of impulsive behaviors. For some individuals, negative self-evaluation can transform into feelings of inadequacy and negative psychological states, resulting in impulsive behaviors (41). Others may respond to negative evaluations with emotions like anger, leading to impulsive aggressive behaviors (42). For athletes, social evaluation anxiety can lead to behaviors such as attacking opponents, negative competition, and dropping out of competitions.

Impulsive behavior is typically influenced by factors such as psychological associations, habits, and sensory stimuli and is often generated unconsciously and difficult to control (43). Self-regulation is defined as the capacity to employ self-control or willpower to manage or counteract impulsive urges (18). It is a goal-directed behavior, and without goal-oriented self-regulation, the process is likely to fail. Attention is one of the key battlegrounds for self-regulation. When individuals notice tendencies toward loss of control due to emotions like anger and self-doubt, timely shifting attention away from the stimulus source helps control impulsive behaviors (44, 45). Therefore, this study presents the following hypotheses:

Hypothesis 4 (H4): Impulsive behavior is positively correlated with social evaluation anxiety.
Hypothesis 5 (H5): Impulsive behavior is negatively correlated with self-regulation.

#### 2.2.3 Mindfulness, impulsive behavior, and mediation

Mindfulness meditation, with centuries of study and practice behind it, forms a critical element of modern functional contextual therapy. There is evidence that mindfulness levels influence impulsivity (46). Impulsive involves a lack of reflection and disregard for consequences. Factors contributing to impulsive behavior include external stimuli, which disrupt emotion regulation, leading to rash behaviors, including aggressive actions. Another factor is self-anxiety and social anxiety (47). Mindfulness can enhance self-regulation capabilities, aligning current actions with their intended purpose, thereby reducing deviations between current actions and ultimate goals. Additionally, mindfulness increases individuals’ acceptance of unpleasant circumstances, reducing the tendency to engage in impulsive reckless actions (48).

Present studies examining the intermediary function of social evaluation anxiety and self-regulation in athletes’ impulsive actions chiefly explore aspects of loneliness (49), happiness (50), and self-recovery (51). For athletes, negative social evaluations are more likely to lead to states of anger, shyness, self-doubt, and self-anxiety, increasing the likelihood of impulsive behaviors on the field (52). Mindfulness can eliminate factors that interfere with self-regulation, enabling athletes to self-regulate as much as possible during competitions (53), reduce their focus on negative social evaluations from the outside, and simultaneously decrease their concern about negative evaluations, focusing on the current competition. This in turn reduces impulsive or violent behaviors caused by loss of self-control and the influence of negative social evaluations (46), reducing biases between current actions and goals (54). Therefore, this study presents the following hypotheses:

Hypothesis 6 (H6): Mindfulness is negatively correlated with impulsive behavior.
Hypothesis 7 (H7): Social evaluation anxiety and self-regulation mediate the relationship between mindfulness and impulsive behavior.

**Figure 1:**
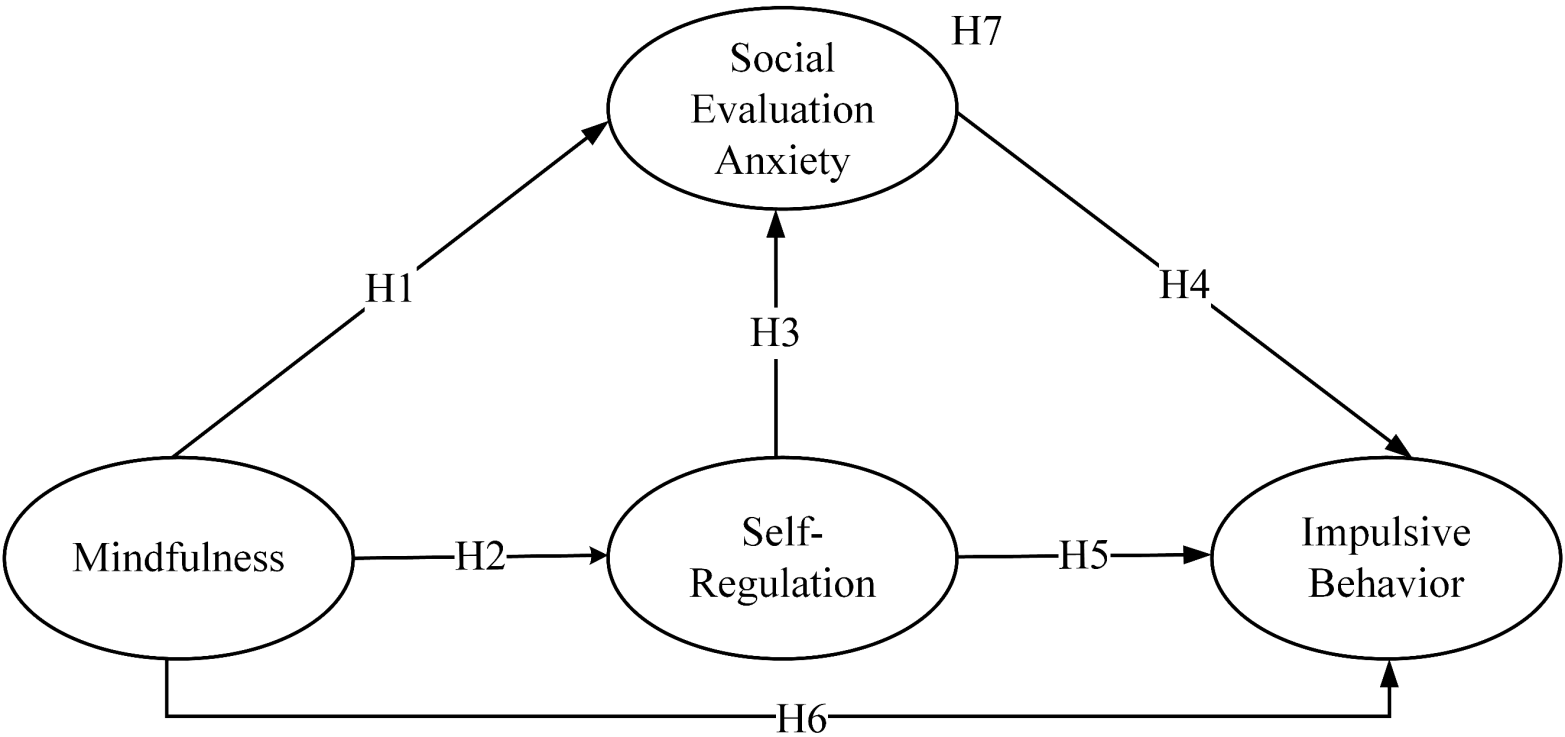
Model overview.

All hypotheses are summarized in **Figure 1**.

## 3 Methodology

### 3.1 Participants and procedures

In this research, an online survey was carried out targeting adolescent athletes who possess specific training experience, utilizing both snowball and convenience sampling techniques. To collect research data, the researchers sent a message in July 2023 to adolescent athletes affiliated with athletic schools, youth training centers, universities, and other institutions in the central China via social networking platforms such as WeChat. The message inquired about their willingness to participate in the study and extended an invitation. If they were willing to participate, the researchers provided them with a link to the questionnaire tool. All participants received information about the survey’s objective and opted to partake voluntarily. Upon finishing the questionnaire, they were rewarded with a small token valued at 10 Chinese Yuan as a gesture of appreciation. After completion, participants were requested to share the questionnaire tool link with their athlete friends and teammates. A sum of 528 questionnaires was disseminated, yielding 403 valid replies, which equates to an effective response rate of roughly 76.3%.

**Table 1** outlines the demographic details of the 403 athletes who took part in the survey.

**Table 1:** Demographic profile of participants.

Profiles		n	%
Gender	Male	260	64.5
	Female	143	35.5
Age	≤18	35	8.7
	18-20	97	24.1
	21-23	146	36.2
	≥23	125	31
Sports items	Football	49	12.2
	Badminton	78	19.3
	Basketball	43	10.7
	Track and Field	64	15.9
	Volleyball	20	5.0
	Swimming	20	5.0
	Gymnastics	23	5.7
	Taekwondo	21	5.2
	Other	85	21
Participation in sports events in the last 12 months	1-3	275	68.2
	4-6	59	14.7
	7-9	23	5.7
	≥10	46	11.4

### 3.2 Instruments

This study’s survey was divided into five distinct sections. The initial segment asked for demographic details from respondents, such as gender, age, the types of sports they participated in, the length of any breaks from training due to sports-related injuries or illnesses within the last year, and the count of sports events they joined in the previous year. The second segment incorporated five questions from a mindfulness scale by Feldman, Hayes (55), aiming to assess the mindfulness levels of participants. Examples of statements include, “I can usually describe my current feelings in great detail.” The third segment drew on four questions from an impulsivity scale by Whiteside and Lynam (56), intended to evaluate impulsive tendencies among respondents. Examples of statements include, “When I feel bad, I often do things that I later regret to make myself feel better.” The fourth segment utilized a scale by Rodebaugh, Woods (57) to assess social evaluation anxiety within the group, with statements such as, “Sometimes I feel like I care too much about what others think of me.” The final segment included five questions from a self-regulation scale by Moilanen (58), aimed at examining self-regulatory behaviors among the participants, with statements like, “When I am sad, I usually go do something that makes me feel better.”

The researchers did not modify the wording of the scale items, but retained only the most relevant items based on the research context, as shown in **Table 2**. To ensure the reliability and validity of the scale, this was followed by a pilot test. Anticipating 97 valid responses, the findings revealed that the Cronbach’s alpha coefficients exceeded 0.8, affirming the thoughtful and context-appropriate modifications to the scales made by the researchers.

**Table 2:** Reliability and validity assessment.

Items	Loadings	Cronbach’s Alpha	CR	AVE
***Mindfulness (MI)***		0.943	0.944	0.770
MI1: It is easy for me to concentrate on what I am doing.	0.881			
MI2: I can accept things I cannot change.	0.840			
MI3: I can usually describe how I feel at the moment in considerable detail.	0.899			
MI4: I try to notice my thoughts without judging them.	0.881			
MI5: I am able to pay close attention to one thing for a long period of time.	0.885			
***Self-Regulation (SR)***		0.930	0.931	0.729
SR1: When I’m sad, I can usually start doing something that will make me feel better.	0.828			
SR2: I can usually act normal around everybody if I’m upset with someone.	0.866			
SR3: I can calm myself down when I’m excited or all wound up.	0.861			
SR4: When I have a serious disagreement with someone, I can talk calmly about it without losing control.	0.838			
SR5: I can stay focused on my work even when it’s dull.	0.875			
***Social Evaluation Anxiety (SE)***		0.949	0.949	0.790
SE1: I am usually worried about what kind of impression I make.	0.858			
SE2: Sometimes I think I am too concerned with what other people think of me.	0.901			
SE3: I often worry that I will say or do the wrong things.	0.917			
SE4: I worry that others will think I am not worthwhile.	0.898			
SE5: I am frequently afraid that I may look ridiculous or make a fool of myself.	0.869			
***Impulsive Behavior (IB)***		0.937	0.937	0.788
IB1: When I feel bad, I will often do things I later regret in order to make myself feel better now.	0.901			
IB2: When I am upset I often act without thinking.	0.862			
IB3: When I feel rejected, I will often say things that I later regret.	0.911			
IB4: Sometimes I do things on impulse that I later regret.	0.876			

### 3.3 Data analysis

In our research, we developed a structural equation model (SEM) utilizing AMOS v23 to explore how mindfulness contributes to diminishing impulsivity among athletes and enhances their impulse control during competitions. We opted for the maximum likelihood (ML) estimation technique for determining the parameters of the model. The analysis proceeded in two phases, initially assessing the reliability and validity of the model, followed by an evaluation of fit indices and path coefficients for the proposed hypothesis and the investigation of mediating effects.

The collected questionnaires have been verified to follow a normal distribution. This finding is crucial as it validates the assumption of normality required for many statistical tests, enhancing the reliability and generalizability of the results. Ensuring that the data adheres to a normal distribution also facilitates more accurate and meaningful interpretations of the statistical analyses performed on the dataset.

To mitigate the risk of common method variance (CMV) linked with self-reporting, we adopted the strategy suggested by Mossholder, Bennett (59). Following this strategy, a side-by-side comparison was made between two models, focusing on changes in degrees of freedom and chi-square statistics. The analysis indicated that the chi-square statistic for the first model was 3861.623, with 152 degrees of freedom, resulting in a p-value less than 0.001. For the second model, the chi-square statistic stood at 373.884, with 146 degrees of freedom, also resulting in a p-value less than 0.001. These results underscore a consistent alignment between the first and second models, suggesting the non-issue of CMV in this study due to the absence of a univariate structure indication.

## 4 Results

### 4.1 Measurement model

The evaluation of latent variables’ reliability and validity incorporated conducting confirmatory factor analysis (CFA) via AMOS v.23. Indicative of the model’s internal coherence, every variable attained Cronbach’s α scores exceeding 0.9 (see **Table 2**), consistent with the standards set by Fornell and Larcker (60). Additionally, each variable’s average variance extraction (AVE) exceeded 0.7 (as presented in **Table 2**), surpassing the established threshold of 0.5. Moreover, the composite reliability (CR) for each latent variable surpassed the 0.9 threshold, offering strong support for the model’s solid convergent validity. The stability of convergent validity throughout the examined models was verified. Principal component factor analysis produced factor loadings ranging between 0.828 and 0.917 (see **Table 2**), highlighting the significant construct validity of the measurement model. Discriminant validity was convincingly established, as evidenced by the square root of the AVE along the diagonal exceeding inter-construct correlations (see **Table 3**).

**Table 3:** Pearson correlation matrix.

Construct	MI	SR	SE	IB
MI	(0.877)			
SR	0.457 **	(0.854)		
SE	-0.373 **	-0.484 **	(0.889)	
IB	-0.416 **	-0.446 **	0.607 **	(0.888)

The square root of the AVE is in diagonals; off diagonals are a Person’s corrections of contracts. **p < 0.01.

### 4.2 Structural model

**Figure 2:**
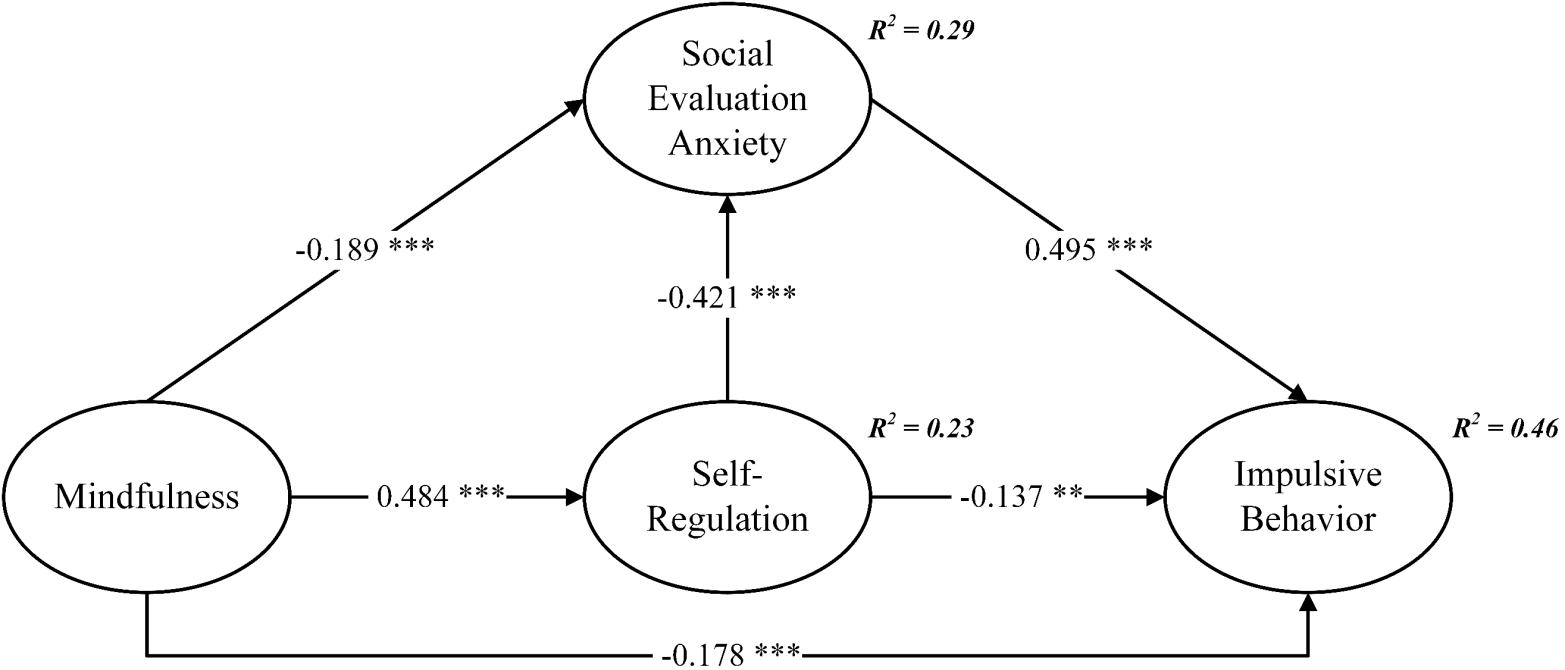
Structural path analysis. **p < 0.01, ***p < 0.001.

After thorough validation of the measurement model’s reliability and validity, this study proceeded to examine the structural model using AMOS v.23, aiming to validate the proposed hypotheses. The confirmatory factor analysis (CFA) outcomes, bolstered by 5,000 bootstrap samples, met established benchmarks (χ^2^/df = 2.561, GFI = 0.907, NFI = 0.950, TLI = 0.964, CFI = 0.969, RMSEA = 0.062), confirming the model’s fit with the data. Pearson correlation analysis reinforced the variable interrelations, as detailed in **Table 3**. The structural model’s standardized coefficients are illustrated in **Figure 2**.

**Figure 2** shows mindfulness negatively correlated with social evaluation anxiety (*β* = -0.189, *p* < 0.001), affirming H1, and positively with self-regulation (*β* = 0.484, *p* < 0.001), supporting H2. Additionally, self-regulation negatively correlated with social evaluation anxiety (*β* = -0.421, *p* < 0.001), backing H3, while social evaluation anxiety positively influenced impulsive behavior (*β* = 0.495, *p* < 0.001), confirming H4. Self-regulation also negatively impacted impulsive behavior (*β* = -0.137, *p* < 0.01), validating H5. Notably, mindfulness’s negative correlation with social evaluation anxiety (*β* = -0.178, *p* < 0.001), further supports H6.

**Table 4** details the mediation analysis conducted with bootstrap estimation, involving 5,000 resamples to determine 95% bias-corrected confidence intervals. Findings highlighted mindfulness’s indirect influence on impulsive behavior via self-regulation and social evaluation anxiety, with a significant estimate of -0.260 (SE = 0.039, CI = [-0.338, -0.187], *p* < 0.001), reinforcing strong evidence for H7.

**Table 4:** Mediation analysis.

	Point Estimate	Product of Coefficients		Bootstrapping		
				Bias-Corrected 95% CI		Two-Tailed Significance
		SE	Z	Lower	Upper	
MI → IB	-0.260	0.039	-6.667	-0.338	-0.187	< 0.001

## 5 Discussion

### 5.1 Theoretical contributions

This research enhances the theoretical understanding of athletes’ impulsive behavior during competitions in various aspects. Primarily, prior studies have concentrated on measuring individual impulsivity (10) and assessing the effects of athletes’ impulsiveness on competition results (3). However, there’s been scant exploration of the links between social evaluation anxiety, self-regulation, and impulsiveness among athletes. This study specifically addresses athletes’ concerns about negative evaluations during competitions and impulsive behaviors triggered by external pressures and provocations. This targeted focus enriches the theoretical research in this domain. The researchers posit that grasping the underlying causes of athletes’ impulsivity in competitions could pave the way for viable solutions. Aligning with prior findings, this study also observes that athletes in contact sports exhibit a higher tendency towards impulsivity (61). Moreover, it extends the discussion by emphasizing that the impulsivity of athletes stems from the stress of negative social evaluations and a deficit in self-regulation abilities. The fear of negative evaluations increases athletes’ anxiety on the field, making them more prone to irritability and depression (42). Inadequate self-regulation skills do not help them overcome this anxiety and regain a favorable competitive state, resulting in mistakes, negative performances, withdrawals, or even a series of aggressive behaviors (62). Furthermore, after the competition, athletes who have experienced defeat face additional negative public criticism, which further increases their psychological pressure, leading them into a state of pessimism and timidity (63). This state greatly impacts their subsequent daily training and psychological pressure for the next competition. The lower level of self-regulation is insufficient to help them quickly recover from this adversity, resulting in a vicious cycle that harms the athletes.

Second, this study delves into the correlations among mindfulness, social evaluation anxiety, and self-regulation. Findings reveal a notable negative association between mindfulness and social evaluation anxiety, a positive link between mindfulness and self-regulation, and a negative relationship between social evaluation anxiety and self-regulation, as depicted in **Figure 2**. These findings support the results of previous studies by Goldin and Gross (32) and Terry and Leary (35). Mindfulness exerts the strongest influence on self-regulation, with social evaluation anxiety following. They both mediate how mindfulness affects impulsive behavior, accounting for 46% of its variance as illustrated in **Figure 2**. This research offers an insightful avenue into exploring the nexus between mindfulness and impulsivity, beginning with athletes’ social evaluation anxiety and examining how it, along with self-regulation, impacts athletes.

However, it should be noted that in our examination of the relationships among mindfulness, social evaluation anxiety, self-regulation, and impulsive behavior, we observed that while some path coefficients fall below the conventional threshold of 0.2, this does not undermine their statistical significance or theoretical importance. These smaller path coefficients likely reflect the complexity and subtlety inherent in psychological constructs. In the fields of psychology and behavioral sciences, the relationships between influencing factors may not be as direct or strong as those in physical or biological processes, thus even smaller effects can hold significant theoretical relevance. Additionally, the magnitude of path coefficients can also be influenced by factors such as sample size, measurement error, and model complexity. Therefore, in this study, although some path coefficients did not reach the empirical standard of 0.2, they still reveal how mindfulness impacts athletes’ impulsive behavior mediated by social evaluation anxiety and self-regulation. This detailed analysis aids in a deeper understanding of how these variables interact and influence athletes’ psychological dynamics.

### 5.2 Practical implications

Given mindfulness positively influences the mitigation of social evaluation anxiety and enhancement of self-regulation (13), and indirectly curtails athletes’ impulsivity in competitions (46), athletes ought to prioritize their mental health. They should strive to enhance their mindfulness levels and strengthen self-regulation skills, allowing them to maintain a stable and positive mindset regardless of the challenges they face during competitions. For example, when confronted with provocations from opponents, athletes can stay focused on the game without letting the opponent affect their emotions (64). When dealing with unfavorable referee decisions and unfair treatment, they can adjust their anger and negative emotions, maintaining a positive attitude (65). In situations where they receive negative comments from others during or after a game, they can shake off feelings of discouragement and respond to malicious criticism and discussions calmly. Moreover, athletes should reflect after competitions to comprehend the causes of their impulsivity, assess its detrimental effects on their performance, and identify effective strategies to prevent such issues in future events.

Additionally, athletes can leverage emerging technologies such as artificial intelligence, immersive technologies, and neurotechnology to enhance their self-control abilities, reducing the negative impacts caused by emotional dysregulation (66, 67). For example, artificial intelligence can be used to create personalized training programs that adapt to the psychological needs of each athlete, predicting stress triggers and suggesting coping mechanisms in real-time. Immersive technologies, such as virtual reality, can simulate high-pressure scenarios common in competition, allowing athletes to practice maintaining composure and mastering emotional responses in a controlled environment. Furthermore, neurotechnology, including neurofeedback and brain stimulation tools, can help athletes gain direct insight into their brain activity patterns associated with impulsivity and anxiety. By training with these tools, athletes can learn to modulate their brain functions, leading to improved emotional stability and enhanced performance under stress. These technologies not only support athletes in mastering technical skills but also empower them to manage their emotions effectively, which is crucial for success in competitive sports. This will help them strengthen their self-regulation and self-control abilities, leading to better performance in the next competition.

For a long time, there has been a misunderstanding of athletes. Due to their robust physicality and superior athletic skills displayed on the sports field, some people perceive them as physically and psychologically strong. When athletes experience emotional fluctuations, they are often judged more harshly, receiving more severe criticism than non-athletes (68). Athletes’ daily training is focused on honing their physical and athletic skills, but during high-intensity training, they often face various psychological challenges. Due to the existence of stereotypes, some athletes may feel ashamed to acknowledge their psychological issues and may try to conceal them. This can lead to further deterioration, resulting in emotional instability, impulsive behavior, and more (69). This study not only identifies how athletes’ exposure to pressure and stimuli during training and competitions can lead to impulsive behavior but also encourages athletes to address their psychological issues. When athletes recognize that they have psychological problems, it is perfectly normal, and they should adjust and seek help promptly. This can be achieved by adopting suitable relaxation techniques, sharing concerns with others to alleviate psychological stress, discussing competition strategies and adjustments with coaches, and contemplating professional mental health support. Having psychological issues is not something to be ashamed of, and addressing them early is crucial to prevent further deterioration, which can significantly impact athletes’ well-being.

### 5.3 Limitations

First, the study did not thoroughly examine potential moderating factors like social anxiety, self-control, and self-efficacy within the modeling framework. Future studies should delve deeper into model diversification and expansion. Second, the cross-sectional nature of this research constrains its comprehensiveness. Subsequent investigations should utilize longitudinal approaches and form experimental control groups. Third, the research employed convenience and snowball sampling techniques, focusing on central China for questionnaire distribution and collection, lacking a broader, national scope. Finally, this experiment collected a larger number of questionnaires from participants involved in sports such as badminton and athletics, potentially biasing the data towards these sports. Future research should strive to include a balanced representation of each sport to ensure more authoritative experimental data.

## 6 Conclusions

Consistent with the aims of this research, findings suggest that athletes exhibit different levels of impulsivity in competitions, linked to shortcomings in emotional regulation abilities, adversely impacting their performance outcomes. Additionally, the research findings reveal the significance of mindfulness, social evaluation anxiety, and self-regulation in influencing athletes’ impulsive behavior. Mindfulness impacts impulsivity via the mediation of social evaluation anxiety and self-regulation. Hence, this research advocates for athletes to integrate mindfulness meditation into their routine training to improve self-regulation skills. This enables athletes to actively regulate their states during competitions, reducing the impact of negative evaluations and external pressures, and maintaining a positive competitive mindset. Furthermore, athletes should acknowledge their psychological issues and seek timely adjustment and, if necessary, professional help. This research not only highlights how athletes’ exposure to pressure and stimuli during training and competitions can lead to impulsive behavior but also encourages them to address their psychological issues proactively, thus maintaining a positive competitive mindset and showcasing their original athletic abilities. Additionally, this study encourages athletes to confront their psychological issues head-on, accept their psychological conditions, and seek help when needed. Having psychological problems is not something to be ashamed of, and addressing them promptly is essential to prevent further deterioration, which can significantly impact athletes.

## Data availability statement

The raw data supporting the conclusions of this article will be made available by the authors, without undue reservation.

## Ethics statement

The studies involving humans were approved by the Ethics Committee of the School of Physical Education of Hunan University of Science and Technology (No. ECBPEHNUST 2022/0012). The studies were conducted in accordance with the local legislation and institutional requirements. Written informed consent for participation in this study was provided by the participants’ legal guardians/next of kin.

## Author contributions

ZZ: Conceptualization, Investigation, Methodology, Writing – original draft, Writing – review & editing. HJ: Investigation, Resources, Writing – original draft, Writing – review & editing. HW: Conceptualization, Project administration, Writing – original draft, Writing – review & editing. YL: Supervision, Writing – original draft, Writing – review & editing.
